# Bibliographical Notices

**Published:** 1849-12

**Authors:** 


### BIBLIOGRAPHICAL NOTICES.

Physician and Patient; *Or a Practical view of the Mutual Duties, Relations and Interests of the Medical Profession and the Community.* By Worthington Hooker, M. D. New York: Baker & Scribner, 1849. pp. 453. 12mo.

Deontologie Médicale: *ou Des Devoirs et Des Droits des Médecins; Dans L'Etat Actuel de la Civilization.* Par le Docteur Max. Simon. A Paris, chez J. B. Bailliere, 1845. pp. 590. 8vo.

Medical Deontology: *Or Concerning the Duties and Rights of Physicians, in the Present State of Civilization.* By Doctor Max. Simon.

*On the Relations of the Physician to the Sick, to the Public and to his Colleagues.* By the late Christopher William Hufeland, M. D. Oxford: 1846. pp. 37. 16mo.

The production of the works whose titles we have just given, are significative and encouraging. They manifest a desire on the part of medical men to investigate their duties and their rights, both to each other and to society at large; and they give promise that the results of such investigation, while quickening the profession to renewed efforts for the discharge of its duties, will inspire it also with proper confidence in support of its rights.

The entire subject is very comprehensive, owing to the numerous and diversified relations of the physician to his fellows, to the community, and to the state. A code of medical ethics, or of medical deontology, as it is called by M. Simon, has a far wider reach than that of regulating the conduct of physicians in their intercourse with each other, and with the sick—important as rules for this purpose undoubtedly are. Its enactments must include, likewise, the direction of the mind to the right studies, suitable preparation for discharging the active duties of the profession, and a continued conscientious investigation of all the facts and phenomena which, either directly or collaterally, make up the science of medicine. Rash conjecture and paradox are not only adverse to medical philosophy, but, also, to medical ethics; they injure by the intellectual obliquity of those who indulge in them, to an equal extent with the boastful promises and empirical arts, evincive of moral obliquity. A physician, who is true to the ethical proprieties of his vocation, has not the privilege of indulging in credulity on the one hand, nor cynical scepticism on the other. Truth in her scientific, not less than in her moral, garb, must ever be before him, as at once a companion and a guide. Ingenuity when not sustained by a rigorous logic, becomes little better than frivolity in him whose knowledge and whose fashion of argument are not used so much to aid science as to dazzle the ignorant.

Qualifications of a high order, on the part of the physician, are demanded, not only for the satisfactory discharge of what may be called his strictly professional duties, but, also, of those which consist in monition and advice to the community on subjects relating to public hygiene and the prevention of epidemic diseases. On these occasions, he must show an acquaintance with the elements of political economy, and, above all, of whatever, in this science, relates to the encouragement of agriculture and the production of the most abundant and healthy food.

Here the duties of the physician become blended with his rights. It is his duty to acquire the most accurate and detailed knowledge of public hygiene in all its bearings. It is also his right to make himself heard, even though he should not be formally consulted, on the application of this knowledge to the preservation of the public health. In this office he performs the part of a practical philanthropist, both by warding off or removing numerous physical ills, and by consequent improvement of public morals; for it is quite obvious that the laws of hygiene are ever in harmony with the moral code, and are necessary both to its adequate comprehension and successful operation. How, for example, can people living in squalid misery, huddled together in small, damp and badly ventilated lodgings, and who, in place of using wholesome food, stupify themselves with alcoholic poison in the shape of distilled spirits; how can their brains be favorably impressed with the lessons of morality and religion; how escape the deteriorating influence of the polluted moral as well as physical atmosphere with which they are surrounded? It is easy to see that the physician, in pointing out proper sanitary measures, and giving advice for establishing an efficient medical police, imparts powerful support to legal enactments for the preservation of the public peace, which is ever most disturbed in the most unhealthy parts of all large cities. He becomes at the same time an efficient coadjutor with the directors of public and the teachers of Sunday schools.

His advice and services in this way must not be viewed in the light of incidental gifts, which can be withheld or offered by him, and received or refused by others, at will. They are, on the contrary, an integral part both of his duties and of his rights.

So, likewise, ought his knowledge of climatic influences, and of medical geography at large, to be freely invoked by government for its guidance in fitting out either maritime or military expeditions, making selections of scites for military posts, encampments, &c. The grief felt by every humane person at the destruction of human life by war, must be greatly increased when it is known, that, for one man lost on the field of battle, at least two men sink under diseases incident to the exposures of a camp life, and faulty military hygiene, arising, for the most part, from a neglect to consult the medical corps on the best means of preventing these disastrous results.

We must, however, on this point, grant that medical men have been two often below the requirements of their station; and that an acquaintance with climatic influences and the causes of endemic diseases—medical geography in its large sense—is too seldom a part of their attainments, and hardly ever included among their positive studies. The neglect is to be the more regretted, and is, at the same time, the less excusable, when we reflect on the admirable model for inquiries of this nature, furnished by Hippocrates himself, who, in this, as in so many other important particulars, added example to precept.

It is not our purpose, at this time, to enter into a minute analysis of the works now on our desk; nor is it necessary critically to examine their respective merits. The Medical Deontology of M. Simon is more æsthetical—the treatise of Dr. Hooker and the tract of the German professor more practical; the former contains more of the objective—the latter of the subjective. We would not be understood, however, to mean that they are broadly contrasted in these respects, or that the French author is deficient in specific applications, or the American and German in general principles. We merely indicate the more obvious tendencies of each of them.

M. Simon begins with an elaborate introduction, in which he exhibits the prominent obligations of a physician to the sick and the community, and the trials and temptations to which he is exposed; and at the same time the author notices some of those higher relations of a physician to the philosophy of life, to political economy, to morals and to religion.

In the first book he treats of the *Duties of Physicians to themselves and to Science,* and discusses in its several chapters the motives by which the physician is governed in the cultivation and practice of medicine; the influence of medical studies, and of the habitual spectacle of suffering, on his mind; the moral and intellectual qualities which he ought to exhibit in the practice of his profession; skepticism in medicine; medical literature; the duties of physicians who engage in the mission of contributing to the advancement of the science and the progress of the art, by the publication of their labours; veracity as a duty towards science; the influence of the passions in the cultivation of the medical sciences; criticism in medicine.

The second book is devoted to an investigation of the *Duties of Physicians to their Patients.* It includes chapters on the general duties of physicians in their intercourse with the sick; also particular duties which they owe to women and aged persons; their duties during the prevalence of epidemic and contagious diseases; therapeutical circumspection; esoteric medicine; measures which ought to be interdicted by physicians in their treatment of the sick, their duties to their patients when diseases are incurable; and to the nervous and hypochondriacal; the limits to which experimenting in medicine ought to be restricted; moral medicine; the use of intimidation in the treatment and prophylaxes of diseases; euthanasy.

In the third book, *on the Duties of Physicians to Society,* M. Simon discusses the following topics: medical skepticism of members of the community generally, and the means by which the physician ought to overcome it; how he ought to combat the dangerous prejudices which he meets with in society; the sinister influence which medical theories may exert on society; medicine considered in its relations to civilization; the subjects on which the physician may enlighten the legislator in the actual state of civilization; how medicine may become the auxiliary of good morals, and compensate in a measure for the imperfection of the law; the general duties of medical legists.

The fourth and last book, on the *Rights of Medicine*, embraces a view of the organization of medicine; medical responsibility; certain immunities which it would be but equitable to grant to physicians, as a compensation for their peculiar burthens; finally, the political rights of physicians.

On all these various themes, M. Simon writes in a lofty and liberal spirit: liberal in its tone of expanded, yet practical benevolence, and lofty in its standard of professional worth, and of professional obligation. Different from too many of his countrymen, he lays down Christian morals as the true basis of medical ethics, that on which the physician can most safely rest the rules for his guidance in his intercourse with the sick, with his fellow practitions, and with society. Thus enlightened, he will never lose sight of his noble mission, so far as to minister to vitiated taste and vulgar prejudices, or to attempt to win his way to public notice by the arts of a paltry trafficker.

We wish it were in our power to follow M. Simon in his successive sketches, and to reproduce the fine literary and moral coloring which he imparts to them; but our narrow limits forbid the attempt, and we must pass on to a notice of the work of Dr. Hooker.

The production of the American author is couched in the same genial spirit as his French contemporary; and we cannot help feeling, while we peruse his pages, that he is himself what he tells us the physician ought always to be, "a hopeful, a cheerful man."

In his introduction, Dr. Hooker tells us, that one object of his book is to expose certain medical errors which constitute the material of quackery, "and to show the *modus operandi* by which the genius of imposture has produced from them the fantastic and ever-changing shapes of empiricism."

Another object of the author is to present the claims of the medical profession to the respect and the confidence of the community. "The confidence reposed on us is not as intelligent as it should be. It is unsettled and capricious."

In his attempt to establish their claims to confidence, he does not lose sight of one great obstacle to success. It is the quackery which is practiced among medical men, and which "is a much greater evil than that which is abroad in the community." He attacks it therefore with no unsparing hand. The author very judiciously remarks: "When the rules of an honorable professional intercourse shall come to be properly understood and appreciated by the public, one of the great sources of the success of quackery will be removed."

The want of a common standard of judgment for the physician and the friends and attendants of the sick, sadly interferes with the purposes and plans of the former. To the elucidation of the difficulties arising from this cause, a considerable portion of the present work is devoted.

When treating of the uncertainty of medicine, and the obstructions encountered in its study and practice, Dr. Hooker lays great and merited stress on the importance of a well educated medical profession.

In the opening chapter, he enumerates the chief causes which make diseases complicated, and, as a consequence, medicine uncertain. The argument makes no pretention to originality; but it is clearly stated under its appropriate heads. Next follow observations on *Skill in Medicine*, which he regards as "*appreciating the condition of the patient in all respects; and applying remedies in the best manner to relieve that condition.*"

*Popular Errors* is the theme of another chapter, which, though most common among the crowd, are not seldom indulged in by the profession. The *post hoc ergo propter hoc*, and a sort of universality attributed to favorite remedies, are errors not confined to the annals of quackery on the one hand, nor of credulity on the other, in which physicians have no participation.

*Quackery* is discussed in chapter fourth, and its grand cause, the referring effects to false causes, pointed out. Quack medicines are, the author thinks, principally of three kinds: evacuants, alteratives, and those supposed to have a specific action on the lungs. The popular pathology of quacks, it might have been mentioned, consists in impurity of the blood, and their therapeutics, in harmony with this view, profess to furnish remedies which are supposed to eliminate or neutralize the peccant matter.

Dr. Hooker mentions the quantities of inert and damaged articles used in the preparation of quack medicines. The certificates in favor of alleged cures by these articles, are chiefly of four kinds: 1. Forgeries; 2. Those which are untrue; 3. Those given by invalids imagining themselves to be relieved; 4. Those given by invalids who are relieved while taking the medicine, and who infer that the relief was obtained by this latter. *Apropos* of certificates of clergymen, it is too truly said: "No class of men have done more harm by giving certificates of cures by quack medicines, than clergymen."

The assistance given to these various systems of imposture, coming under the general head of quackery, by the newspaper press, is also very great. "Not content with advertising quack medicines, they, [the newspapers,] for a liberal fee, admit into their columns, articles which have the appearance of editorial recommendations; and these are copied, as such, into advertisements in other newspapers. And besides all this, respectable [?] editors have often refused to publish any exposure of the impositions of quackery."

The author next describes other forms of quackery: such as itinerant lecturers, who sell books, medicines, trusses, breathing tubes, &c. "Some of these empirical lecturers have, as a special attraction, one or two lectures, particularly for the ladies, to which no gentleman can be admitted; and one or two, also for gentlemen, from which the ladies are excluded. I will only say," continues Dr. Hooker, "that in every case in which I have known this to be done, the character of the lectures has been such as no virtuous community should tolerate."

Animal magnetism, as applied to medicine, is passed in review by the author, who also notices in the same connection Paracelsus, St. John Long, and Perkins with his metallic tractors. Some amusing instances of credulity and boastful promise are given in relation to this last mentioned phase of quackery, or Perkinism, as it was called, which, in less than ten years after the summing up of the five thousand cures, was only thought of as a thing that was past. The far-famed tractors are now almost forgotten.

*Thompsonism,* or *Thomsonianism,* next comes in for a share of critical notice and commentary. The prominence which this system of qnackery once acquired, and still in some quarters retains, may be a justifiable cause for repeating, under the title of its principles, a farrago of assertions in contradiction to anatomy, physiology and common experience. We have not ourselves the patience to repeat even the outlines of such a tissue of absurdities, notwithstanding that Thompson has been put, by Dr. Bartlett, on the same line of theorists as Dr. Rush.

*Homœopathy* is the subject of chapter sixth, and forms one of the examples of a wide spread and, in reference to the pecuniary gains of many of those who practice it, successful quackery. After an outline of the theory of Hahneman, Dr. Hooker enumerates the causes of its *apparent* success; among which faith, and a strict regard to diet and regimen, must be considered the chief ones. An occasional stealthy use of remedies in ordinary use, also explains in part some of the cures obtained.

Unsound and untenable in theory, homœopathy is impossible, if the practice is attempted to be carried out according to the dicta of infinitesimal doses; and it is an imposture if, under the plea of these doses, those in common use or of appreciable quantity and strength are used. Its advocates may take their choice, they must either assume an impossibility, nothing short of continual miracles, if they are honest in their belief, or they must practice or connive at fraud, in having recourse to medication of a different nature from that which they profess.

Chapter seventh is taken up with *Natural Bone Setters*, and their manipulations, which sometimes reduce an old dislocation, but more frequently cause a laceration of ligaments, and aggravate chronic inflammations of joints. Of their blunders in this way, Dr. Hooker furnishes some striking proofs.

Under the head of *Good and Bad Practice*, in chapter eighth, we meet with many judicious reflections on the fallacy which is fostered by taking results as proofs of good practice, without a knowledge of all the antecedent and associate circumstances of the case. To determine the difference between good and bad practice, requires, 1st, " a sufficient amount of evidence; 2d, skill in observation."

Dr. Hooker protests against the common belief " that a man may be an ignoramus in regard to other subjects, and yet may have great skill in medicine." On the contrary, he very correctly alleges, that " true skill in the practice of medicine, requires the possession of a wide range of talents, and among them sound judgment or (as it is familiarly called when used in reference to ordinary subjects,) common sense is pre-eminent."

The author furnishes " five ways of judging of the skill and attainments of the physician."

" The reader has seen that there are then *five* ways of judging of the skill and the attainments of a physician. 1st. By examining his opinion on medical subjects, and the reasons upon which they are based. 2d. By observing his practice, and comparing its results with those of the practice of others. 3d. By inquiring into the evidences of his education. 4th. By observing the unbiassed opinions entertained of him by his medical brethren. 5th. By observing his mental qualities as they are exhibited in relation to those subjects which the observer himself understands.

If the inquirer be a physician, he can very properly make use of the two first-named means of arriving at the estimate. But if he be a non-professional observer, he must for the most part give up these means, as being liable to lead him into error, and resort to the remaining ones. That he should *entirely* give up the two first means I do not claim. All that I claim is, that he should place very little reliance upon them, while his chief reliance should be upon the three last."

The reason why intelligent men have not availed themselves of those means of forming a correct opinion on so important a subject, are set forth in this chapter, in pages 237–9. They are, chiefly, the contented selfishness of a large number of the medical profession, who are satisfied with the false position which they themselves have acquired, by arts resembling those of the quack; and in part by another class, whose intentions were at first good, but who despairing of reform, yield too readily to the current of imposture, if they do not actually exert themselves to keep in it.

A lesson of wisdom is, however, now being taught to these recreants from true science and philanthropy, which may amend them by its acting directly on the sensitive part of their nature.

" They find that their cunning subservience to the false opinions of the people, has increased the hold of those opinions upon the public mind ; and, as a wide door has thus been opened for quackery, they find that the same arts, in using which they have been so successful, are now used quite as dexterously by the whole herd of ignorant quacks and showy pretenders. They find that the Homœopathist is stealing away some of their best, and, as they thought, their most reliable patients. The Thompsonian, the Chronothermalist, &c., are committing similar depredations. And of all this they have no right to complain, because these pretenders obtain these patients by the same artful and deceptive means, by which these physicians at first acquired them, and by which they have so long retained them heir patrons."

It is a melancholy truth, repeated by Dr. Hooker, that the profession itself has given birth to much of the quackery of the present day.

In the ninth chapter, under the head of *Theory and Observation*, Dr. Hooker points out the true basis of theory; consisting as it does, in observation, and the facts which furnish materials to the observer. The mortifying remark is made, that the history of medicine is very much a history of theories. We have, however, the consolation to know, as mentioned by the author, that there is a reform in medicine in progress,—a breaking loose from theory, and an adherence to rigid observation.

In the following chapter, having for its subject the *Popular Estimates of Physicians*, we read of the generally erroneous estimate made of them, and the means by which the community may be enabled to form a more correct judgment. Too true is the remark, that medical education is practically despised by a large portion of the public. Indeed, so far is this carried, that the very acknowledgment by the world, of a physician being a close student and a learned man, is made in a tone and manner which imply disqualification for the successful practice of his profession. In the main, we may receive the test of the possession by the physician of certain necessary mental qualities, laid down by Dr. Hooker, viz: to observe them as exhibited by him, in regard to subjects which the observer understands in common with the physician. We say in the main; we ought to have added, with the proviso that the observer, who forms his judgment of the physician, has himself an adequate fund of common sense, and an ability to make a fair comparison, and draw a correct conclusion. There are so many men whose ideas do not extend beyond their tools, their looms, their weights and measures, and their yard stick, that only a comparatively small number remain to enact the part of judges of professional qualities and merits.

The *Means of Removing Quackery* constitute the subject of chapter eleventh. The author, after alluding to faulty efforts to undeceive the public on this subject, points out those which he deems to be more efficient. Among these latter are the individual influence of physicians, especially exerted on the intelligent who sustain quackery; and careful abstinence, by medical men, of either directly fostering the evil by certificates in its favor, or of giving it countenance by their own empirical proceedings. That there is a strong spirit of quackery in the medical profession, the tendency to which must become every day greater, with the rapid and disproportioned increase of its numbers, is, we fear, too evident, unless stringent rules be enforced, by enactments and agreements emanating from its own body. The author regards the formation of the American Medical Association an important movement in the destruction of quackery. We are sanguine enough to entertain similar hopes, with the understanding that the profession comes forth in adequate numbers, and that its voice is neither stifled, nor made to utter false notes, by cliqueism, or by attempts to confine, from year to year, executive action in the same small circle.

In the chapter on *Intercourse with Physicians*, we meet with much sound and discriminating inquiry. The following remarks convey a salutary hint even to those who have joined various associations in a spirit of good fellowship, and with kindly, if not benevolent intentions.

"The disposition to jealousy and strife in the medical profession is also promoted by the associations which are sometimes formed by physicians with each other, or with the community, for the sake of furthering their own selfish ends. Professional cliques on the one hand, and alliances with various societies, social, moral or religious, on the other, when relied upon as means of advancing one's professional interests, are always inimical to the harmony of medical men. They render competition unfair and dishonorable, and therefore contentious. The physician who calls to his aid the influence of a sect, or a party, or an association of any sort, in so doing not only places himself in an attitude to awaken distrust, but subjects himself, in maintaining the alliance, to a necessity for employing means of self-aggrandisement, which will conflict with the rights of others, and will therefore involve himself in either a secret or an open warfare with his brethren."

*The Mutual Influence of Mind and Body in Disease* is a theme too vast to be fully discussed in the single chapter devoted to it by Dr. Hooker. The aspects under which he examines it, are chiefly ethical, as governing the physician in his deportment in the sick room. While, in general, it is the duty of the medical attendant to do his best to soothe and allay all nervous and mental excitement, he will sometimes find it useful to encourage this state, and especially by appeals to the imagination, and by diverting the mind from attention to the bodily feelings as well as to common daily cares.

Chapter fifteenth, on *Insanity*, contains a pleasing outline of this important subject, exceedingly useful to the general, and not without interest even to the professional reader.

Connected with the subject of the chapter on the mutual influence of mind and body, is that of chapter sixteenth, on *Hope*. The nice question, of how far hope is to be nourished in the mind of a patient, and how far the truth, melancholy as it may be, should be told, is examined on the present occasion by Dr. Hooker, in this and in the following chapter, on *Truth in our Intercourse with the Sick.*

The evils of deception are pointed out in two classes of persons, in whose case it is too often supposed to be a venial error, if not actually justifiable. These are children and the insane. Both of them are keener observers, and have a better perception and recollection of right and wrong practised upon them than is generally imagined.

The *Moral Influence of Physicians* is a fertile subject. The peculiar and intimate relations of the physician with the Sick, his acquaintance with all their infirmities, both of body and mind, and the unlimited trust so often reposed on him, under these circumstances, require the greatest circumspection and delicacy, as well in speech as in manner. His influence on the mind weakened by disease, or yet plastic in the young and inexperienced, is great, and in its exercise subjects him to a dread responsibility. With the various details of quarrels and bickerings in families, and between individuals, whose medical adviser he is, he has nothing to do, except (and the exception is indeed an important one) to soothe angry feelings, and to be a mediator and peace maker. This does not imply his becoming an arbitrator, the office of which demands more time, and leads to investigations which sometimes increase existing difficulties, and subject him to the danger of being a party to the dispute, in place of a disinterested friend intent in allaying it.

*Trials and Pleasures of a Medical Life* are the subjects of the nineteenth and concluding chapter, of "Physician and Patient." They give a picture in which, although the shading must predominate, there are still gleams of sunshine, and bits of bright landscape, and lively groups, which, if they do not excite to joy, at least enable us, every now and then, to throw off sadness and inspire us with renewed hopes in our pilgrimage.

The reader will have seen from the preceding outline, meagre as it is, the scope and aim of Dr. Hooker in writing this work. We cannot forbear to add the expression of our pleasure at the successful manner in which the author has performed his task. His train of argument and illustrations are sound and logical; his facts apposite, and the purpose and the style in which the whole is dressed, are in harmony with the subject, and well adapted to secure the continued attention of his readers. We hail the appearance of "Physician and Patient" as a valuable addition to our medical literature, in a field which is but just opening to our view, but in which an abundant harvest will yet be reaped, for the common benefit of the profession, who will gather it in, and of the authors who have sowed the seed.

We shall conclude with a few passages taken from the Advice of Hufeland. We have called this little work a tract, and so it is in size, object and mode of publication. It is extracted from the "*Enchiridion Medicum, oder Anleitung zur Medicinischen Praxis*" of the author, and is issued in an English dress, in its present form, by Dr. Greenhill, of Oxford. This last named gentleman, whose classical lore excites admiration, and whose earnest piety commands our reverence, has given a portion of his valuable time to the editing of small volumes of the biography of medical men, eminent for the religious faith, and of Letters and Addresses, together with prayers, with a design to encourage religion and piety in the profession generally.*

---

* Among these (published in Oxford and London) we find the lives of George Cheyne and Thomas Harrison Burder, and of the Rev. Sir James Stonhouse, Bart., M. D., Burder's Letters from a Senior to a Junior Physician, Address to a Medical Student, and Prayers for the use of the Medical Profession; making in all six volumes, 16mo. To these we believe we may add as of subsequent publication, although we have not seen them, the Life of Dr. Thomas Willis, and the Life of Sir Thomas Browne. Dr. Greenhill gives a list of twenty-four physicians, most of them of admitted eminence in the profession, either as writers, authors, or practitioners, respecting whom, he asks to be furnished with Letters and Papers, &c. "The profits of these little works, if any, will be given to some Medical charity."

The Relations of the Physician, as laid down by Hufeland, are threefold; 1. Towards the sick; 2. Towards the public; and 3. Towards his colleagues. The obligations of the physician under these several heads are so well and clearly inculcated, that, on perusing them, we felt tempted to transfer the contents of the little volume entire to our pages; but we must reluctantly refrain. We cannot, however, dismiss the subject without introducing to the notice of our readers some of the portions of the advice of the learned and amiable author.

After speaking of the *attention*, *accuracy* and *conscientiousness* demanded of the Physician, he adds the following picture of what a medical man ought to be in his address, manner and general deportment.

"But skill and art alone are not sufficient. He must be particularly mindful of his *conduct*. It is this which recommends him to the public, and creates confidence and admittance; for, as the generality of people are incompetent to pronounce on his science, it is natural for them to take their measure of his ability from the measure of his conduct. By force of conduct alone a Physician of very moderate talents may become the favorite of the public, and without it the most skilful professional man remain unnoticed and unappreciated. Of his external appearance also he must not be regardless; it should comport with the dignity of his station and the importance of his duties.—In the main features of his conduct he should be apt to create confidence, friendly with dignity, decent without affectation, gay but not frivolous, serious when he ought to give importance to his subject and his words, complaisant and indulgent in all trifling matters, but firm while executing important measures and sustaining the pronounced sentence: sympathizing and cordial, of sound sense and regard for Religion and its consolations, neither taciturn nor loquacious, much less a newsmonger, but devoting his whole attention to the sick, noticing every circumstance, careful in the examination of the patient, observing even those around him, neither eccentric nor vulgar, neither coxcomb nor pedant, but holding to the middle way in all things; especially not passionate and angry, but calm and circumspect; for a quiet and sober sense creates confidence.—It is a great fault common to young practitioners, particularly of late, that they strive principally to excite sensation, whether it be by the newest fashion of dress or science, or by love of paradox and singularities, or even by charlatanism.

But there is a great difference between exciting sensation and creating confidence; yea, the former prevents the latter from taking place, and it is only by the latter that a lasting prosperity is founded. Exciting sensation can, of course, have the effect of making a Physician the topic of conversation for some time, and even of procuring him a large concourse of patients, but the attraction of novelty soon ceases, and the meteor vanishes into nothingness. On the other hand, the silently meritorious practitioner, honestly persevering and unwearied, may remain for a time unnoticed, but, in establishing him slowly in the love and confidence of the better part of his neighbors, he lays a surer and firmer foundation for future prosperity."

On the policy not less than the duty of kindly bearing and speech of a Physician towards and respecting his professional brethren, we read these emphatic lines.

"Oh! that I were able to impress the minds of my brethren with the truth as forcibly as I am penetrated by it, that he who degrades a colleague, degrades himself and his art. For, in the first place, the more the public becomes acquainted with the faults of Physicians, the more will Physicians become exposed as contemptible and suspicious, and the more will such exposure impair confidence; and, when confidence in the whole body is diminished, each member, and the censurer included, will lose a share of it. The public would be less prone to censure the medical profession, and its faults would not be a favorite topic of conversation, if the members themselves did not broach it, and set the bad example. It shews a short-sighted selfishness and want of all common spirit, when a Physician acts in such a manner, and thereby hopes to raise himself in the same proportion as he degrades others. Further, such conduct is in opposition to the first principles of morals and Religion which command us, not to lay bare the faults of others, but to overlook and excuse them."

The following "*Maxims and Rules for Beginning Practitioners*" terminate this little tract,' and most worthily too both for the author and his editor.

"Every sick person is a temple of Nature: approach it with awe and devotion, devoid of frivolity, egotism and want of principle; then will Nature look at you with grace, and disclose to you her secret.

Bear always in mind, who you are and what your office is. You are employed by God as priest of the holy flame of life, and as administrator and distributer of His highest gifts, health and life, and of the secret powers which He has bestowed throughout Nature for the benefit of mankind. A sublime, a sacred task! Perform it purely; not for your advantage, nor for your fame, but for the glory of the Lord, and the salvation of your fellow man. You will one day have to give an account of it.

Maintain always the dignity of the profession in yourself and in others; and never degrade it so as to make it a trade, and the means of bad purposes.

In imminent danger to the sick you must risk all, even reputation.

In general, never think of yourself, but of the patient.

The most sublime vocation of man, after the service of the Deity, is that of being priest of the holy vital flame, and an administrator of God's highest gifts, and of the most secret powers of Nature—in one word, a *Physician.*

Do you think, that, when you appear before the throne of Eternal Truth, you will be asked, "After what system did you act? Did you comply with it, and have you brought it to perfection?" The question will be this: "I made you steward of the wonderful powers which I have placed in Nature and her products to benefit mankind; how did you distribute those treasures? for the benefit of mankind, with gratitude and adoration? or for the honor of your name, with selfishness and egotism? Did you, in your researches and actions, strive merely for truth, to help your brethren, or was it all for self-interest?"

To him, to whom Medicine becomes not a Religion, it is the most disconsolate, troublesome and ungrateful art on earth; yea, it must become with him the greatest frivolity and sin; for only that which is done in God, is holy and beatifying. How is it now-a-days with many? Nothing but a mere speculation, a means to make a fortune, to gain money and notoriety: even with the better sort of practitioners, the pursuit of the healing art reaches no higher than an investigation of Nature."

---

*The three kinds of Cod Liver Oil; comparatively considered with reference to their Chemical and Therapeutical properties.* By L. J. De Jongh, M. D., of the Hague. Translated from the German, with an Appendix and Cases, by Edward Carey, M. D. To which is added an article on the subject from "Dunglison on New Remedies." 8 vo. pp. 211. Lea and Blanchard, Philadelphia. 1849.

Although Cod Liver Oil was employed in the treatment of disease by Percival some seventy-five or eighty years ago, and subsequently recommended by a number of writers, through medical journals and tracts, as a valuable remedy for various complaints, it can hardly be said to have been extensively employed until within a few years past; and now, there is scarcely another article in the list of the Materia Medica which receives so large a share of the attention of physicians. Gold, antimony, mercury, sarsaparilla, iodine, &c., which have been regarded, in their day, as the great modifiers of nutrition, and, therefore, in some sort as specifics in various chronic and cachectic diseases, seem almost by common consent to have been laid aside for this new eutrophic. Every thing that can shed light on a subject of such general interest must be acceptable, and although there have been numerous papers devoted to it recently, the present publication is undoubtedly the most full and complete exposition of the history of Cod Liver Oil, embracing its preparation and therapeutic properties, that we have yet seen.

It would seem probable from Dr. De Jongh's investigations, that the three kinds of cod liver oil which supply the German and English markets, are derived from the same source, and owe their differences to the circumstances of preparation.

In the United States, we have also three varieties of the oil, viz. brown, yellow, and pale—principally, however, the brown and the pale—and it is quite probable that their differences depend upon similar causes, although there is ground for believing that the same fish do not supply the European and American markets. In the former—at least in England and Germany—the Bergen oil is officinal; and this, according to the author's correspondents, who seem to have the best means of knowing, is chiefly supplied by the livers of the Dorse (Gadus Carbonarius sive Aselus Niger;) while that employed in this country is obtained from the livers of the Torsk (Gadus Morrhua sive Aselus Major.) But, it is alleged that the fishermen are not very particular in selecting the precise species, and that some half a dozen are used indiscriminately for the production of cod liver oil. As far as we have been able to discover, the oil procured from these different species presents no great differences in composition or in therapeutic effects, so that the experiments performed with the Bergen oil, apply also to that generally used in the United States.

We have not space for a detailed account of the very elaborate quantitative and qualitative analyses of the author, but the following is his "*general summary.*"

100 *parts of Cod Liver Oil contain,*

| | Brown | Light Brown. | Pale. |
|---|---|---|---|
| Oleic acid with brown substance (Gaduine, and two peculiar bodies | 69.78500 | 71·75700 | 74·03300 |
| Margaric acid | 16·14500 | 15·42100 | 11·75700 |
| Glycerine | 9·71100 | 9·07300 | 10·17700 |
| Butyric acid | 0·15875 | ... | 0·07436 |
| Acetic acid | 0·12506 | ... | 0·04571 |
| Fellic and cholic acids, with some oleine, margarine, and bilifulvin | 0·29900 | 0·06200 | 0·04300 |
| Bilifulvin, and bilifellinic acid, and two peculiar substances | 0·87600 | 0·44500 | 0·26800 |
| A peculiar substance, insoluble in alcohol of 30° | 0·03800 | 0·01300 | 0·00600 |
| A peculiar substance, insoluble in water, alcohol and ether | 0·00500 | 0·00200 | 0·00100 |
| Iodine | 0·02950 | 0·04060 | 0·03740 |
| Chlorine with some bromine | 0·08400 | 0·15880 | 0.14880 |
| Phosphoric acid | 0·05365 | 0·07890 | 0.09135 |
| Sulphuric acid | 0·01010 | 0.08595 | 0·07100 |
| Phosphorus | 0·00754 | 0·01136 | 0·02125 |
| Lime | 0·08170 | 0·16780 | 0·15150 |
| Magnesia | 0·00380 | 0·01230 | 0·00880 |
| Soda | 0·01790 | 0·06810 | 0·05540 |
| Iron | a trace | ... | ... |
| Loss | 2·56900 | 2·60319 | 3·00943 |
| | 100·00000 | 100·00000 | 100·00000 |

"If we compare these results with those which the analysis of the Cod Liver Oil by Spaarmann and Marder have afforded, it will be seen that the green soft resin and brown hard resin, the reddish yellow viscous substance, the peculiar coloring matter, and the phocenic matter of the older authors are wanting; whilst these again recognize neither the component parts of the bile, the acetic and butyric acids, nor the peculiar brown substance (gaduine.)"

At first, it was supposed that the therapeutic effects of Cod Liver Oil arose altogether from the iodine contained in it—more lately, however, it has been believed that iodine is not a constant constituent, and not necessary for the proper action of the oil. But Dr. De J. alleges that from his investigations "it invariably follows that the real Bergen Cod Liver Oil always contains iodine; not, however as free iodine, because by the mere carbonization of the oil without saponification no iodine was detected even in the purest Cod Liver Oil. When, however, after the method of saponification no iodine whatever is detected, it is impossible that it can be pure Bergen oil." The author maintains that where the article is genuine, if no iodine be found by the process he has laid down, it is because it has been removed by the means employed to bleach it for market; and that "the difficulty of discovering iodine can only arise from the employment of a wrong process, or of an impure oil."

By a "comparison of the three kinds of Cod Liver Oil, it is shown, first, that the lighter kinds are richer in inorganic substance (as well as in iodine), than the brown kind; whilst, on the contrary, the latter is richer in the component parts of the bile, butyric and acetic acids. In general, the light-brown and the pale oil agree in every respect, much more than the brown, by which it is established that the light-brown is only a pale oil which has become old."

In order to determine the relative value of the three kinds of oil as curative agents, Dr. De J. instituted experiments with them in various diseases, from which he discovered that the cures with the brown Cod Liver Oil were in general effected in half the time required by the two other sorts, and hence he ascribes to it more powerful healing properties.

"This difference in its operation can only be attributed to some variation in their chemical relations." His concluding remarks contain his explanation of this point.

"That the Cod Liver Oil is composed of different substances has been sufficiently proved by chemical analysis. Neutral fat, biliary matter, iodine, phosphorus, all substances of acknowledged power, as well as butyric acid, gaduine, &c.: lastly, the many organic salts which are found therein.

The question naturally arises, to which of these different constituent parts of the Cod Liver Oil is its efficacy owing? Whether to the iodine, or the fat, or the phosphorus, or to the others. These questions are solved with great difficulty; for in all diseases in which the Cod Liver Oil is found to be efficacious, the physician has to fulfil many indications simultaneously, if he expects the perfect restoration of his patient. In general, the weakened digestion is to be corrected, the secretions must be restored, and the lymphatic system brought to a higher state of activity; besides which, and what seems to be the most important of all, the tone of the nervous system is to be improved. The slightest reflection will show that neither biliary matter, nor the fatty substance, nor the iodine, nor any one single constituent part of the Cod Liver Oil, is able, of itself, to fulfil these indications; consequently its power as a remedy is not to be ascribed exclusively to one only, but to the united operation, if not of all, still of the greater part. It is not, as is the case with the quinine in the Peruvian bark, that there exists a peculiar active principle in the Cod Liver Oil, but as each individual constituent part fulfils a peculiar indication, it is possible that the operation of the whole is successful in the cure of the diseases.

Nevertheless, I do not think that all the constituent parts are equally powerful; on the contrary, some of them possess greater efficacy: and it is, in every case, those which fulfil the most important indication that are to be the most esteemed. I now come to the comparison of my observations.

The brown Cod Liver Oil has proved itself a most powerful remedy in rheumatism and scrofula. Now, I have found, in my chemical analysis, a quantitative difference between this and the other sorts of Cod Liver Oil. The constituent parts, which exist in great proportions in the brown Cod Liver Oil, must, therefore, be considered as those which fulfil the most important indications. The neutral fat, the iodine, phosphorus, the inorganic salts, exist in the same proportions in the other kinds of oil, which are not supposed to be particularly efficacious in scrofula and rheumatism; it is, therefore, fair to assume that the brown Cod Liver Oil owes its greatest power to the biliary matter and butyric acid, which exist in it in much larger proportions than in the lighter-coloured oils."*

In conclusion, I have still to remark that the substance, unknown until now, which I first found in the product of the Gadus tribe, and called Gaduine, should, by no means, be considered as possessing that peculiar active principle; on the contrary, I am inclined to think that, on account of its perfect insolubility—in the form, at least, in which I found it—it is altogether inactive."

The author's experience with Cod Liver Oil in phthisis, scrofula, and rheumatism, is highly encouraging. In reference to the former of these diseases, he remarks:

"If we compare the opinions of different persons in different places, which in the main all agree with what we have already said on the subject, we can form a pretty correct judgment on the virtues of the Cod Liver Oil in tubercular phthisis. This medicine may be called the grand restorer of health; for the successful operation of any remedy whatever against so formidable a disease, may indeed deserve that appellation. Should the disposition to phthisis be present, the use of Cod Liver Oil will prevent the further development of the disease, sometimes permanently; generally, however, for a considerable period. Whereas, when it is given in the already developed form of phthisis, although the perfect cure cannot be accomplished, life will be prolonged and rendered more supportable. This is, indeed, all that can be expected from any remedy in the present imperfect state of our knowledge of tubercular phthisis."

* Klencke also mentions the importance of the biliary matter, without even knowing of its existence in the Cod Liver Oil; for he declares the yellow Cod Liver Oil to be a substitute for bile. We cannot, however, agree with Klencke's opinion on the transformation of the oil into albumen.—*German Trans.*

